# Sepsis impairs microvascular autoregulation and delays capillary response within hypoxic capillaries

**DOI:** 10.1186/s13054-015-1102-7

**Published:** 2015-11-05

**Authors:** Ryon M. Bateman, Michael D. Sharpe, Justin E. Jagger, Christopher G. Ellis

**Affiliations:** Department of Medical Biophysics, University of Western Ontario, London, ON Canada; Department of Biochemistry, Keio University School of Medicine, Tokyo, Japan; Ryleeon, 18519-77 Ave NW, Edmonton, AB T5T6A8 Canada; Department of Anesthesia and Critical Care Western, University of Western Ontario, London, ON Canada

## Abstract

**Introduction:**

The microcirculation supplies oxygen (O_2_) and nutrients to all cells with the red blood cell (RBC) acting as both a deliverer and sensor of O_2_. In sepsis, a proinflammatory disease with microvascular complications, small blood vessel alterations are associated with multi-organ dysfunction and poor septic patient outcome. We hypothesized that microvascular autoregulation—existing at three levels: over the entire capillary network, within a capillary and within the erythrocyte—was impaired during onset of sepsis. This study had three objectives: 1) measure capillary response time within hypoxic capillaries, 2) test the null hypothesis that RBC O_2_-dependent adenosine triphosphate (ATP) efflux was not altered by sepsis and 3) develop a framework of a pathophysiological model.

**Methods:**

This was an animal study, comparing sepsis with control, set in a university laboratory. Acute hypotensive sepsis was studied using cecal ligation and perforation (CLP) with a 6-hour end-point. Rat hindlimb skeletal muscle microcirculation was imaged, and capillary RBC supply rate (SR = RBC/s), RBC hemoglobin O_2_ saturation (SO_2_) and O_2_ supply rate (qO_2_ = pLO_2_/s) were quantified. Arterial NOx (nitrite + nitrate) and RBC O_2_-dependent ATP efflux were measured using a nitric oxide (NO) analyzer and gas exchanger, respectively.

**Results:**

Sepsis increased capillary stopped-flow (*p* = 0.001) and increased plasma lactate (*p* < 0.001). Increased plasma NOx (*p* < 0.001) was related to increased capillary RBC supply rate (*p* = 0.027). Analysis of 30-second SR–SO_2_–qO_2_ profiles revealed a shift towards decreased (*p* < 0.05) O_2_ supply rates in some capillaries. Moreover, we detected a three- to fourfold increase (*p* < 0.05) in capillary response time within hypoxic capillaries (capillary flow states where RBC SO_2_ < 20 %). Additionally, sepsis decreased the erythrocyte’s ability to respond to hypoxic environments, as normalized RBC O_2_-dependent ATP efflux decreased by 62.5 % (*p* < 0.001).

**Conclusions:**

Sepsis impaired microvascular autoregulation at both the individual capillary and erythrocyte level, seemingly uncoupling the RBC acting as an “O_2_ sensor” from microvascular autoregulation. Impaired microvascular autoregulation was manifested by increased capillary stopped-flow, increased capillary response time within hypoxic capillaries, decreased capillary O_2_ supply rate and decreased RBC O_2_-dependent ATP efflux. This loss of local microvascular control was partially off-set by increased capillary RBC supply rate, which correlated with increased plasma NOx.

**Electronic supplementary material:**

The online version of this article (doi:10.1186/s13054-015-1102-7) contains supplementary material, which is available to authorized users.

## Introduction

The microcirculation is a highly integrated and functional system [1] that delivers oxygen (O_2_) and nutrients and removes waste products and heat from cells, thereby maintaining cell function and making the microcirculation essential for muscle and organ function. The microcirculation has distinctive architecture, with the skeletal muscle microvasculature investigated in this study consisting of feeding arterioles, capillary networks and collecting venules. A hallmark of sepsis is an early onset microvascular dysfunction, within 6–24 hours in animal models, characterized by increased capillary stopped-flow and a maldistribution of microvascular blood flow [2–4]. De Backer et al [5] were the first to report that outcome in septic patients was related to small vessel perfusion density in the sublingual microcirculation and more recent studies have underscored the importance of microvascular dysfunction in multiple organ failure and patient mortality [6–8].

However, while previous experimental studies have implied an impairment of microvascular autoregulation [2–4, 9], this is not completely understood. Moreover, the effect of sepsis on the capillary response within hypoxic capillaries (micro-tissue regions with low capillary red blood cell (RBC) hemoglobin O_2_ saturation (SO_2_) and low tissue oxygenation) is unknown. The significance is that impairment of the microvascular autoregulatory system would uncouple local O_2_ delivery from local O_2_ demand leaving some tissue regions vulnerable to hypoxia, and possible dysfunction. Evidence suggests the microcirculation can modulate regional capillary flows via erythrocyte O_2_-dependent ATP signaling from hypoxic RBCs [10, 11]. Our working hypothesis is that ATP released from hypoxic RBCs [12, 13], via a deoxy-hemoglobin/glycolytic enzyme molecular switch at the inner RBC membrane [13–15], can bind to purinergic type 2 (P2Y) receptors on endothelial cells and trigger a conducted vascular response [9] via endothelial cells to upstream resistance vessels, which respond via nitric oxide (NO)-mediated modulation of vascular tone resulting in increased downstream RBC supply rate [10, 16, 17]. Whether this RBC function is altered during sepsis is unknown.

Accordingly, in this study of the early onset effects of sepsis on microvascular function, we considered two related but unknown aspects of the microvascular autoregulatory system. First we measured the microvascular in vivo capillary response time within hypoxic capillaries (capillary RBC SO_2_ < 20 %) at the arteriolar and venular end of the skeletal muscle capillary network and second we tested the null hypothesis that sepsis would not alter RBC O_2_-dependent ATP efflux. We then incorporated these findings into a multifactorial model of microvascular pathophysiology based on current evidence.

## Methods

### Animal model of sepsis

Experimental protocols were approved by the University of Western Ontario Council on Animal Care. Sepsis was studied using a saline fluid resuscitated, hypotensive animal model as previously described [2]. The study design was a comparison between two groups undertaken in a University setting. In brief, 11 male Sprague-Dawley rats were divided randomly into sham/control and cecal ligation and perforation (CLP) groups. Sepsis was induced in anesthetized animals by perforating the cecum and expressing the fecal contents into the peritoneal cavity. Animals were cannulated for fluid resuscitation (0.9 % saline, 18 mg.kg^−1^.hour^−1^), monitoring mean arterial pressure and blood collection. A tracheotomy was performed for mechanical ventilation with fraction of inspired O_2_ = 0.3. Core temperature was maintained at 36.5–37.2 °C. The right hind limb extensor digitorum longus skeletal muscle was isolated and repositioned into the optical path. Animals were stabilized and microvascular images acquired from 4–6 hours after the septic injury. See Additional file 1 for data supplement and detailed description.

### Blood samples and NOx, lactate and RBC O_2_-dependent ATP analysis

Arterial blood was collected to establish normal blood gases at the outset and again at 6 hours for NOx (NO_2_^−^ + NO_3_^−^), lactate and ATP efflux analysis. NOx was measured using a NO analyzer as previously described [18, 19]. RBC O_2_-dependent ATP efflux was measured using a custom gas exchanger. In brief, arterial whole blood was equilibrated under normoxic (N) then subjected to hypoxic conditions (H), for 5 minutes respectively, as previously described [13]. ATP efflux was normalized as the H/N ratio. See Additional file 1 for data supplement and detailed description.

### Functional microvascular imaging

A dual wavelength imaging system acquired optical density (OD 420, 430 nm) information from the skeletal muscle microcirculation, as previously described [3, 20]. In brief, capillary RBC supply rate (SR = RBC/s) was calculated from RBC velocity and lineal density measurements [3], and RBC SO_2_ was calculated from the OD430/420 ratio [21]. Capillary oxygen supply rate (qO_2_) was then calculated from RBC SR and SO_2_, where qO_2_ (pLO_2_/s) = SR × SO_2_ × k, where k = 0.0362 pL O_2_/RBC at 100 % SO_2_ [3]. Heterogeneity in RBC SR and qO_2_ was calculated as the coefficient of variation (SD/mean) from 30-second profiles. Random fields of view were imaged and recorded. During off-line analysis, a three-line reference grid was used to quantify functional capillary density (caps/mm), as either continuous, intermittent (RBC flow came to arrest at least once) or stopped-flow (arrested RBC flow) based on 30-second analysis of flow behavior [22]. Capillary response time was assessed as the time required to restore RBC SO_2_ to >20 %. See Additional file 1 for data supplement and detailed description of capillary hemodynamics and RBC SO_2_ measurements, see Additional file 2 for a video clip of capillary RBC hemodynamics and see Additional file 3 for a video clip of capillary RBC SO_2_ measurements.

### Statistics

All values are reported as mean ± SE unless otherwise stated. *P* values less than 0.5 were considered statistically significant. Comparisons between CLP and sham group variables were made using the student’s *t*-test or Mann–Whitney Rank Sum test. Linear regression was used to test the relationship between capillary RBC SR and plasma NOx. Chi-squared analysis was used to test the null hypothesis that no difference in capillary O_2_ supply distribution (low, average, high) existed between sham and CLP. SigmaStat 3.0 software (Point Richmond, CA, USA) was used for statistical analysis.

## Results

**Fig. 1:**
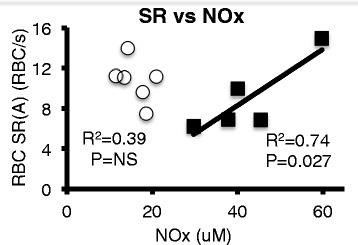
Relationship between capillary red blood cell (RBC) supply rate (SR) and plasma NO_2_^−^ + NO_3_^−^ (NOx). RBC supply rate (RBC/s) at the arteriolar end of capillaries in hind limb skeletal muscle was measured using a functional microvascular imaging system, as described in the Methods. Plasma NOx was measured in arterial blood samples, as described in the Methods. Linear regression detected a significant relationship between capillary RBC supply rate and plasma NOx level in septic animals (*black boxes*). *White circles* = sham

Acute physiological responses to septic injury are shown in Table 1. CLP animals had normal respiratory gases, decreased mean arterial pressure and decreased pH (*p* < 0.001). Though hemoglobin was higher in the CLP group, hemoglobin remained in the normal range (11.5–16.1 g/dL) in both groups. Both plasma metabolites, lactate and NOx (oxidized metabolites of nitric oxide, NO_2_^−^ + NO_3_^−^) increased in CLP animals by the 6-hour end-point (*p* < 0.001; Table 1). Regression analysis found increasing arterial plasma NOx levels were related to increasing capillary RBC SR (*p* = 0.027; Fig. 1).

**Table 1:** Physiological parameters at 6-hour end-point

Variable	Sham (n = 6)	CLP (n = 5)	p value
Weight (g)	162 ± 3.3	164.5 ± 1.5	NS
Cardiovascular and blood gases			
Mean arterial pressure (mmHg)	103.6 ± 2.9	68.8 ± 3.9	<0.001
Arterial PO_2_ (mmHg)	97.6 ± 5.4	108 ± 2.2	NS
Arterial SO_2_ (%)	94.1 ± 2.5	92.7 ± 1.5	NS
Arterial pCO_2_ (mmHg)	37.5 ± 2.9	34.9 ± 2.9	NS
pH	7.43 ± 0.01	7.32 ± 0.03	<0.001
Hemoglobin (g/dL)	11.8 ± 0.3	14.9 ± 0.2	<0.001
Plasma metabolites			
Lactate (μM)	1.1 ± 0.1	2.1 ± 0.1	<0.001
Arterial NOx (μM)	18.1 ± 1.5	42.6 ± 4.1	<0.001

Values are mean ± SE. Normal rat hemoglobin (11.5–16.1 g/dL). *CLP* Cecal ligation and perforation, *NOx* NO_2_^−^ + NO_3_^−^, *NS* Nonsignificant, *PCO*_*2*_ Partial pressure of carbon dioxide, *PO*_*2*_ Partial pressure of oxygen, *SO*_*2*_ Oxygen saturation

### Capillary RBC SR and qO_2_

Variation in capillary RBC SR (RBC/s), qO_2_ (pLO_2_/s) and their respective 30-second coefficients of variation, at arteriolar and venular ends of capillary networks, are shown as box plots for each animal in Additional file 4: Figure S3. Confidence intervals (95 %) for control capillary RBC SR and qO_2_, used to categorize RBC SR (as slow, average, fast) and qO_2_ (as low, average, high) in all experiments are shown in Additional file 5: Table S1. The relationships between capillary qO_2_ and RBC SR in single sham and CLP experiments are shown in Additional file 6: Figure S4. Table 2 summarizes the RBC SR and qO_2_ data at the arteriolar and venular ends of capillaries. While no significant differences in mean capillary RBC SR were detected at either the arteriolar or venular ends of capillary networks, there was a trend (*p* = 0.092) towards increased variation in venular end capillary RBC SR in CLP animals. However, capillary oxygen supply rates were found to decrease at both arteriolar and venular ends of capillary networks (*p* = 0.002) and have more variability (measured as the coefficient of variation) in their 30-second signal. Of note is that some extremely fast capillary RBC supply rates and high oxygen supply rates were detected in some animals (Additional file 4: Figure S3).

**Table 2:** Capillary perfusion, O_2_ transport, functional capillary density, capillary and RBC function

Variable	Sham (n = 6)	CLP (n = 5)	*p* value
Capillary perfusion/O_2_ transport			
RBC SR variation			
art SR (RBC/s)	10.8 ± 0.9	9.0 ± 1.6	NS
art SR (RBC/s) CV (%)	43.1 ± 4.3	48.8 ± 2.4	NS
ven SR (RBC/s)	9.7 ± 1.5	7.6 ± 1.1	NS
ven SR (RBC/s) CV (%)	42.8 ± 5.7	58.6 ± 7.6	=0.092
qO_2_ variation			
art qO_2_ (pLO_2_/s)	20.7 ± 1.2	14.4 ± 2.1	=0.013
art qO_2_ (pLO_2_/s) CV (%)	55.4 ± 5.8	76.4 ± 6.5	=0.024
ven qO_2_ (pLO_2_/s)	16.9 ± 1.9	8.3 ± 1.1	=0.002
ven qO_2_ (pLO_2_/s) CV (%)	59.7 ± 6.0	79.3 ± 8.1	=0.054
Capillary O_2_ ER (%)	19.1 ± 8.0	39.1 ± 7.7	=0.102
Functional capillary density^a^			
CDcontinuous (caps/mm)	20.8 ± 1.7	15.2 ± 0.8	=0.014
CDintermittent (caps/mm)	3.6 ± 1.1	5.8 ± 1.1	NS
CDstop (caps/mm)	3.6 ± 0.4	8.7 ± 1.1	=0.001
Capillary function^b^			
art response time (SO_2_ < 20 %)	2.1 ± 0.3	7.5 ± 0.9	<0.001
ven response time (SO_2_ < 20 %)	2.6 ± 0.7	6.4 ± 1.6	=0.026
RBC function			
RBC ATP efflux (H/N)^c^	1.48 ± 0.10	0.55 ± 0.04	<0.001

Values are mean ± SE

^a^Evaluated on the basis of 30-second flow behavior (intermittent flow = capillary comes to arrest for at least 1 second; stopped-flow (stop) = RBCs are arrested for 30 seconds)

^b^Capillary response time = time required for capillary RBC SO_2_ to return to values >20 %

^c^ATP efflux where H/N is RBC ATP efflux ratio under normoxic (N = RBC exposure to 5 minutes 21 % O_2_) and hypoxic (H = RBC exposure to 5 minutes 0 % O_2_) conditions

*art* Arteriolar end of capillary network, *CD* Capillary density, *CLP* Cecal ligation and perforation, *CV* Coefficient of variation (= SD/mean; based on 30-second RBC SR and RBC qO_2_ profiles), *ER* Extraction ratio, *qO*_*2*_ Capillary oxygen supply rate, *RBC* red blood cell, *SO*_*2*_ Oxygen saturation, *SR* Supply rate, *ven* Venular end of capillary network

The significance is that some regions of the CLP skeletal muscle microcirculation had fast RBC supply rates 9–18 times faster with higher oxygen supply rates supplying from 17 to 26 times more O_2_ than slower capillaries, while other capillaries with stopped-flow were no longer delivering O_2_ to local tissue. Consistent with an average drop in qO_2_ across the capillary bed, there was a trend towards a two-fold increase in capillary O_2_ extraction in CLP animals (*p* = 0.102) compared to sham (Table 2). In addition to changes in capillary RBC hemodynamics and oxygen supply rates, functional capillary density was dramatically altered as continuous flow decreased and capillary stopped-flow increased 2.4-fold (*p* = 0.001; Table 2). See Additional file 1: Figure S2 for a labeled image of the septic microcirculation and Additional file 2 for the corresponding video clip.

### Capillary 30-second SR–SO_2_–qO_2_ profiles

**Fig. 2:**
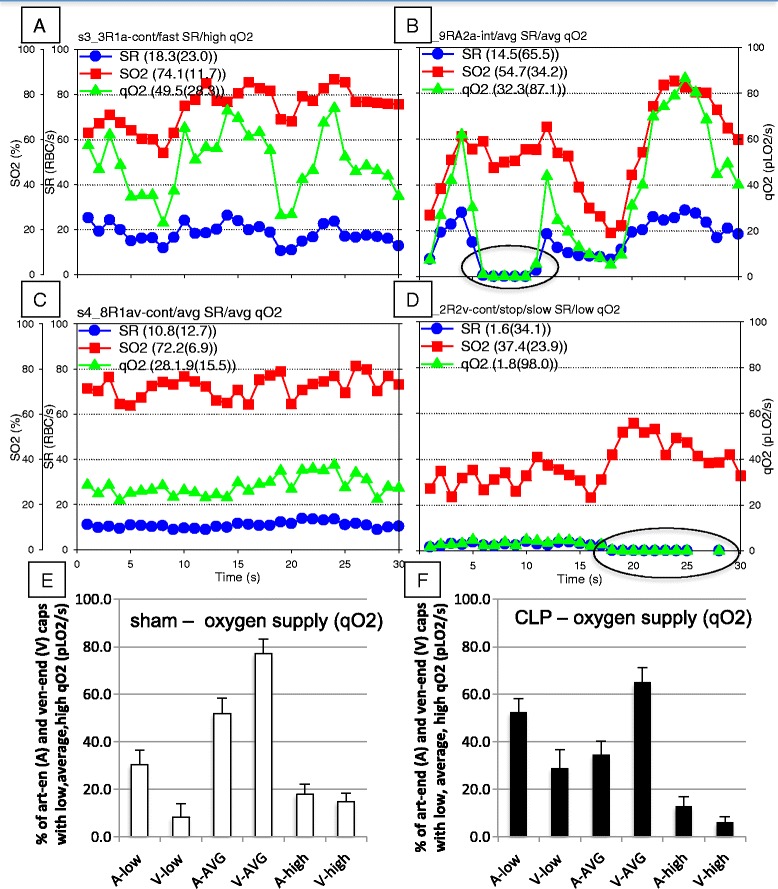
Capillary red blood cell (RBC) supply rate, O_2_ saturation and capillary O_2_ supply rate profiles. Four representative 30-second profiles of capillary RBC supply rate (SR, *blue circles*), RBC hemoglobin O_2_ saturation (SO_2_, *red squares*) and O_2_ supply rate (qO_2_, *green triangles*) are shown in panels **a**–**d**. Based on 95 % confidence intervals (see Additional file 5: Table S1 in sham animals), SR was assessed as either slow, average or fast, while qO_2_ was assessed as being either low, average, or high. Capillaries were also categorized according to their functional capillary density, as either continuous (RBC SR >0 for 30 seconds, panels **a**,**c**), intermittent (if RBC supply rate came to arrest for at least one second, panel **b**, where oval marks interval of stopped-flow) or stopped (if RBCs remained at arrest, panel **d**, provided RBCs remained at arrest for 30 seconds). **e**,**f** qO_2_ distributions for sham and cecal ligation and perforation (CLP) animals, respectively. Legend for 30-second profile statistics (mean (coefficient of variation)). A significant difference between qO_2_ distributions was determined by Chi-squared analysis, *p* < 0.05

Variations in the patterns of capillary RBC SR, hemoglobin SO_2_ and qO_2_ are shown in a series of 30-second SR–SO_2_–qO_2_ profiles (Fig. 2a–d). Each capillary SR–SO_2_–qO_2_ profile was categorized as having (slow, average, fast) SR and (low, average, high) qO_2_. For example, Fig. 2a depicts a capillary with continuous fast SR (18.3 RBC/s) and high qO_2_ (49.5 pLO_2_/s), while Fig 2b and c show average profiles, yet have distinct differences in SR and qO_2_; where the flow behavior in Fig. 2c is continuous, while it is intermittent in Fig. 2b. Figure 2d depicts a capillary with slow SR (1.6 RBC/s) and low qO_2_ (1.8 pLO_2_/s). Distributions of capillary oxygen supply rates in sham and CLP groups are shown in Fig. 2e, f. Chi-squared analysis (*χ*^2^ = 83.7, 5 df, *p* < 0.05) indicated that differences in qO_2_ existed between groups, reflecting an increase in low oxygen supply rate in capillary networks in CLP animals. Thus the septic microcirculation became more heterogeneous in terms of local O_2_ delivery with increased numbers of capillaries having low qO_2_ or no O_2_ delivery at all (in the case of stopped-flow capillaries) and much higher oxygen supply rates in other capillaries.

### Capillary response time within hypoxic capillaries

**Fig. 3:**
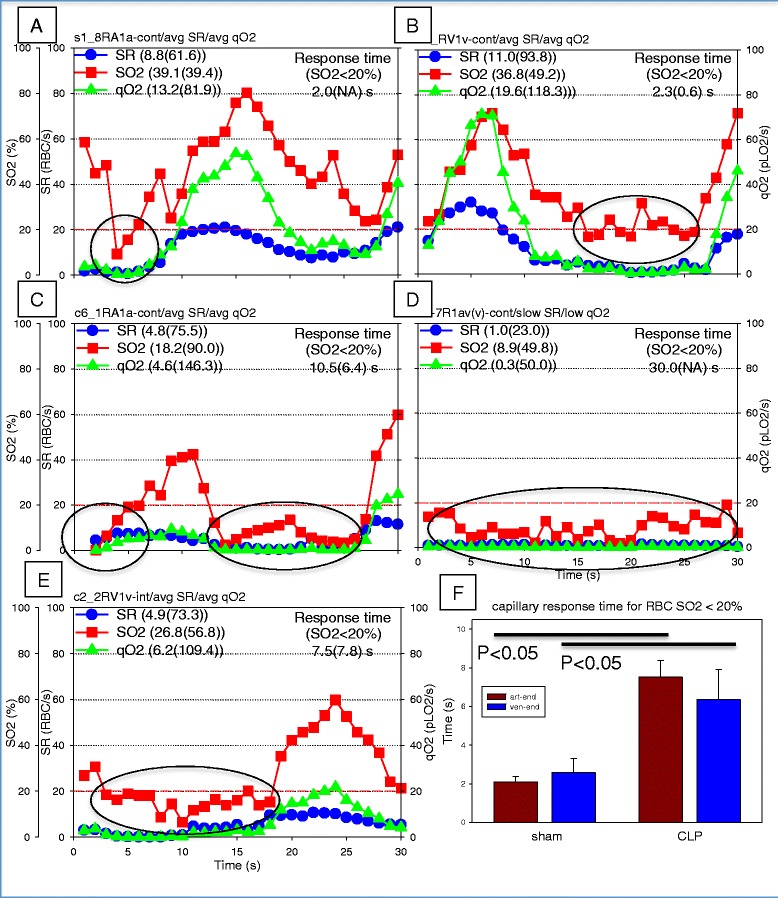
Capillary response time within hypoxic capillary (red blood cell oxygen saturation <20 %). **a**–**e** Five 30-second capillary red blood cell (RBC) supply rate (SR, *blue circles*), RBC hemoglobin O_2_ saturation (SO_2_, *red squares*) and O_2_ supply rate (qO_2_, *green triangles*) (SR–SO_2_–qO_2_) profiles. Capillary response time with low RBC saturation (SO_2_ < 20 %) was assessed as the time required for a capillary to return to a state where RBC SO_2_ > 20 %. The dashed horizontal red line shown in SR–SO_2_–qO_2_ profiles, panels **a**–**e**, at SO_2_ = 20 % is the threshold used to quantify the response. Ovals indicate time intervals in the SR–SO_2_–qO_2_ profiles where capillary RBC SO_2_ had fallen below 20 %. **f** The capillary response times at both the arteriolar (art-end) and venular (ven-end) ends of capillary networks in sham and cecal ligation and perforation (CLP) animals. Legend with profile statistics (mean (coefficient of variation)), capillary response time is mean (SD). *NA* not applicable

Analysis of 30-second SR–SO_2_–qO_2_ profiles revealed that some capillaries experienced a delayed response to periods of low capillary RBC SO_2_ (<20 %, referred to as capillary hypoxia). The capillary response time was defined as the time required for a capillary to return to a state where RBC SO_2_ > 20 %. For example, Fig. 3a, b shows relatively short response times within capillaries with falling capillary RBC SO_2_ (2.0 and 2.3 seconds, respectively), whereas Fig. 3c, e show much longer response times (10.5 and 7.5 seconds, respectively), while Fig. 3d shows a capillary failing to respond with RBC SO_2_ < 20 % over the 30-second observation period. Overall, 2.5- and 3.6-fold increases in capillary response times to RBC SO_2_ < 20 % were detected at the arteriolar and venular ends of septic capillaries (*p* < 0.05; Fig. 3f). The response times are summarized in Table 2.

### Sepsis reduces RBC O_2_-dependent ATP efflux

**Fig. 4:**
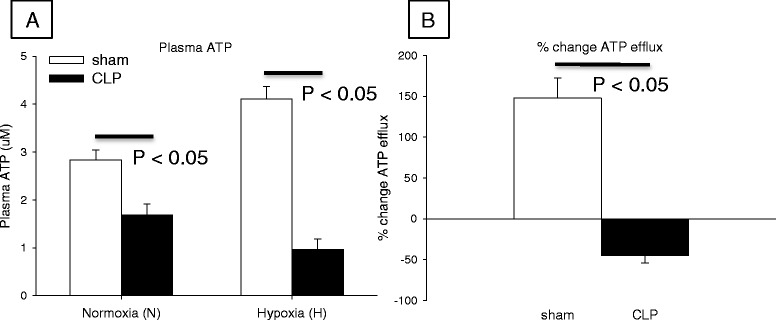
Erythrocyte O_2_-dependent ATP efflux decreased in sepsis. **a** Plasma ATP levels under normoxic (red blood cell (RBC) exposed to 5 minutes 21 % O_2_) and hypoxic conditions (RBC exposed to 5 minutes 0 % O_2_) in sham and cecal ligation and perforation (CLP) animals. **b** The % change in RBC O_2_-dependent ATP efflux. ATP efflux was normalized as the hypoxia/normoxia ratio (H/N) and is summarized in Table 2

Since this study found evidence of delayed capillary response times within hypoxic capillaries, we tested the null hypothesis that sepsis would have no effect on the RBC response to hypoxic conditions by measuring RBC O_2_-dependent ATP efflux. We found RBC O_2_-dependent ATP efflux was impaired in septic RBCs, compromising the erythrocyte’s ability to respond to hypoxic conditions. Under normal physiology, there was a large increase in ATP released from RBCs exposed to hypoxia compared to the normoxic or baseline condition. During sepsis, however, RBCs released much less ATP when exposed to hypoxia (Fig. 4a, b), measured as plasma ATP and % change in ATP efflux, respectively. Expressed as the hypoxia/normoxia ratio, which normalizes the measurement to baseline, we found erythrocyte O_2_-dependent ATP efflux decreased in CLP animals (62.6 % versus sham; 1.48 ± 0.1 versus sham 0.55 ± 0.06, *p* < 0.001), summarized in Table 2 as RBC function.

### Model of biophysical and metabolic factors controlling microvascular autoregulation under normal and septic conditions

**Fig. 5:**
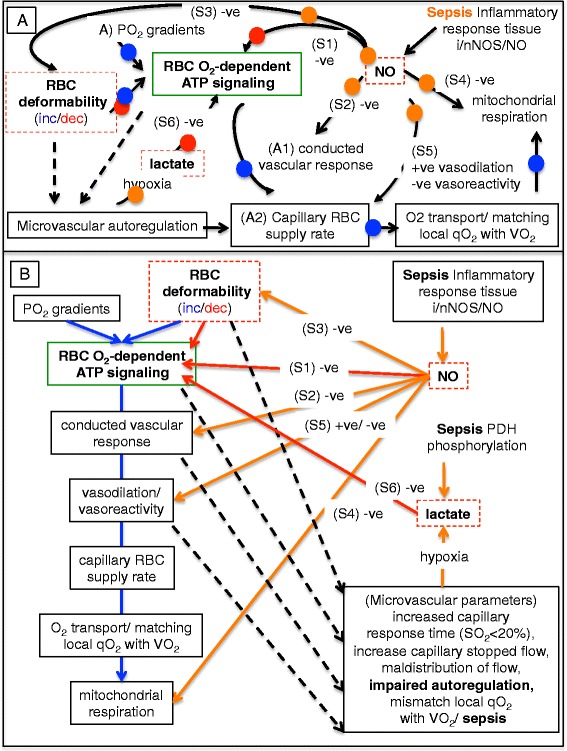
Model of microvascular autoregulation. **a** Pathways involved in microvascular autoregulation. At the model center (*green square*) is red blood cell (RBC) O_2_-dependent ATP efflux, where RBCs act as signal transducers responding to local O_2_ gradients, shear stress and metabolic conditions. *Blue dots* (A–A2) indicate normal microvascular function whereby partial pressure of oxygen (PO_2_) gradients or RBC deformation [36–38] induce RBCs to release ATP, triggering a conducted vascular response leading to increased capillary RBC supply rate [10, 11, 16], matching local O_2_ delivery with demand. *Red dots* indicate negative feedback on RBC O_2_-dependent ATP efflux by nitric oxide (NO) [24], lactate [43] and decreased RBC deformability [42] (*dashed red boxes*). Multiple effects of NO on microvascular autoregulation, O_2_ transport and O_2_ consumption (*orange dots* (S1–S6)) include: S1, inhibiting RBC O_2_-dependent ATP efflux [24]; S2, reducing conducted vascular response [9, 25]; S3, decreasing RBC deformability [22]; S4, inhibiting mitochondrial function [26] and O_2_ consumption [2]; S5, inducing vasodilation, but altering vasoreactivity by inducing arteriolar hyporesponsiveness [28–30]. Sepsis increases lactate (S6) via tissue hypoxia or pyruvate dehydrogenase (PDH) phosphorylation [31], which decreases RBC O_2_-dependent ATP efflux. Sepsis impaired microvascular autoregulation is manifested by increased capillary response time within hypoxic capillaries, attenuated RBC O_2_-dependent ATP efflux, increased capillary stopped-flow [2, 4, 5] and low capillary venular end O_2_ supply rates. **b** A flow chart of the model, where *blue arrows* trace normal microvascular autoregulation, *red arrows* show negative feedback on RBC O_2_-dependent ATP efflux and *orange arrows* indicate NO-mediated effects. *Dashed lines* show relationships to microvascular function and autoregulation during sepsis. *i/nNOS* Inducible/neuronal nitric oxide synthase, *qO*_*2*_ Capillary O_2_ supply, *VO*_*2*_ O_2_ consumption

**Fig. 6:**
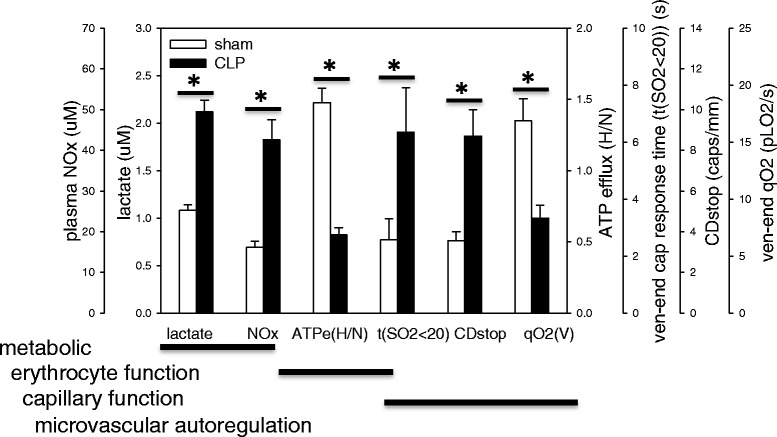
Metabolic, erythrocyte and microvascular changes at the 6-hour end-point. The figure summarizes the metabolic, RBC and microvascular functional changes observed at the 6-hour end-point in this study. *ATPe(H/N)* RBC O_2_-dependent ATP efflux ratio (hypoxia/normoxia; see methods for description), *CDstop* Capillary density of stopped-flow capillaries, *CLP* Cecal ligation and perforation, *NOx* Nitrite + nitrate, *t(SO*_*2*_ *< 20)* Capillary response within hypoxic capillaries, where t is the time required for a capillary to return to a state where RBC oxygen saturation (SO_2_) >20 %, *qO*_*2*_ *(v)* Venular end RBC O_2_ supply rate. *p < 0.05

**Fig. 7:**
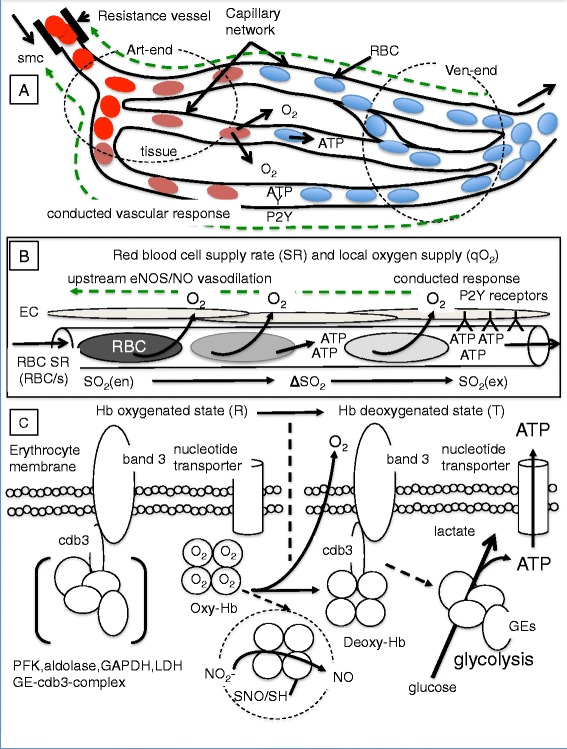
Model of three-levels of microvascular autoregulation. **a**–**c** Schematics of three levels of microvascular autoregulation: 1) the overall skeletal muscle capillary network, 2) the capillary and 3) the erythrocyte, respectively. **a** Microvascular autoregulation at the capillary network level is viewed as the integrated conducted vascular response over the entire network feeding back to the resistance vessels [45], where nitric oxide (NO) relaxes smooth muscle vasodilating feeding arterioles, causing downstream increases in capillary red blood cell (RBC) supply rate (SR) [10, 11]. The *dashed green line* represents the conducted vascular response. **b** At the capillary level is the interaction between vascular ATP (released by hypoxic RBCs) and purinergic type 2 (P2Y) receptors on endothelial cells, which trigger the conducted vascular response. **c** At the level of the hypoxic erythrocyte is the interaction (metabolic switch) between deoxyhemoglobin and cdb3 at the inner RBC membrane, where deoxyhemoglobin displaces glycolytic enzymes, triggering glycolysis and ATP release [13–15, 57, 58]. (Note, RBC O_2_-dependent ATP release is inhibited by glycolytic inhibitors, CO [13] and NO [24]). The *dashed circle* in **c** shows two additional RBC mechanisms. While NO_2_^–^ has been reported to function in hypoxic vasodilation whereby deoxyhemoglobin converts NO_2_^–^ to NO [46, 56], its role in sepsis is unclear. Similarly, it is unclear how hemoglobin-derived S-nitrosothiol [59] would function as a vasodilator in the capillary network, as capillaries are not surrounded by smooth muscle. *Art-end* Arteriolar end of capillaries, *cdb3* Cytoplasmic domain of band 3, *eNOS* endothelial nitric oxide synthase, *GE* Glycolytic enzymes, *Hb* Hemoglobin, *LDH* Lactate dehydrogenase, *NO*_*2*_^*–*^ Nitrite, *PFK* Phosphofructokinase, *qO*_*2*_ Capillary oxygen supply rate, *R* Relaxed Hb state, *Smc* Smooth muscle cell, *SO*_*2*_ Oxygen saturation, *T* Tense Hb state, *Ven-end* Venular end of capillaries

An objective of this study was to incorporate new findings on impaired microvascular autoregulation into a pathophysiological model to gain insight into the mechanisms and possible feedback loops underlying the microvascular derangements observed in skeletal muscle during sepsis. The model (Fig. 5a) simplifies this complex pathophysiology by presenting a simple framework and shows the main interactions under consideration, while limiting the model to three important negative modulators of RBC O_2_-dependent ATP efflux: 1) decreased RBC deformability, 2) increased lactate and 3) increased NO (which is upregulated by inducible nitric oxide synthase (iNOS) in skeletal muscle [2] in this model). At the center of the model is the erythrocyte acting as an O_2_ sensor [17, 23] responding to local partial pressure of oxygen (PO_2_) gradients and shear stress-induced changes in RBC deformability. Also included in the model are a number of related NO and sepsis-mediated microvascular autoregulation, O_2_ transport and O_2_ consumption effects including impaired RBC O_2_-dependent ATP release [24], inhibition of endothelial conducted vascular response [9, 25], loss of RBC deformability [22], inhibited mitochondrial function [26, 27] and decreased skeletal muscle O_2_ consumption [2], and increased vasodilation and altered vascular reactivity [28–30]. Additionally, sepsis increases plasma lactate via tissue hypoxia or phosphorylation of pyruvate dehydrogenase [31], which can feedback on the RBC O_2_-dependent ATP efflux. Figure 5b represents the model as a flow chart. Figure 6 summarizes the metabolic, RBC and microvascular functional changes observed at the 6-hour end-point of this study. Figure 7 is a summary figure that extends the model concept to three levels of microvascular autoregulation including: 1) the overall capillary network (Fig. 7a) where the conducted vascular response is integrated over the capillary network, 2) the capillary (Fig. 7b) where hypoxic RBCs release ATP into the vasculature triggering the conducted vascular response via endothelial cell P2Y receptors, and 3) the RBC (Fig. 7c) where deoxyhemoglobin displaces glycolytic enzymes at the inner RBC membrane triggering O_2_-dependent ATP efflux.

## Discussion

### Summary

The main finding of this study was that sepsis impaired microvascular autoregulation during the initial stages of the septic injury. This was evident in two ways: 1) at the capillary level, we found a three- to fourfold delay in capillary response time within hypoxic capillaries (RBC SO_2_ < 20 %) and 2) at the RBC level, we detected a significant impairment in the ability of septic RBCs to release ATP in response to hypoxic conditions. Both of these findings are consistent with a loss of microvascular autoregulation. In the context of sepsis, this may be important because impairment of microvascular autoregulation may lie at the center of microvascular dysfunction and be an important factor in multiple organ failure by fundamentally altering local tissue O_2_ transport properties, as well as delivery of nutrients, antioxidants and elimination of waste products.

The data reported here suggest there is an uncoupling of local O_2_ delivery from local O_2_ demand leaving some tissue regions vulnerable to hypoxia and unable to rapidly respond to O_2_ demand; this is consistent with Lam et al. [4], who found septic skeletal muscle had a weaker microvascular response to electrical stimulation and increased O_2_ demand than control. This impairment of microvascular autoregulation and capillary O_2_ delivery may, however, be partially offset by increased NO production [2] and local vasodilation, as skeletal muscle capillary RBC supply rate was found to correlate with increasing plasma NOx levels in this study. Previously, we detected an upregulation of iNOS within skeletal muscle, increased NO within the RBC, and increased NOx within plasma and septic skeletal muscle, 3–6 hours after septic injury [2, 22]. As NO levels increase within septic tissue, we suspect one target is smooth muscle cells surrounding the arterial resistance vessels. The resulting vasodilation would increase blood flow in these vessels causing downstream increases in capillary RBC supply rate in capillaries that remained patent. Taken together with the finding that iNOS can inhibit cNOS (where constitutive NOS is associated with microvascular autoregulation) [32], we hypothesize that a trade off occurs between local autoregulatory control of O_2_ delivery at the microvascular level and a more general increase in flow as vascular resistance falls in sepsis. In skeletal muscle there is also evidence of increased capillary fast flow as sepsis progresses out to 24 hours [3], suggesting fast flow may be a later response to an earlier loss of functional capillary density and microvascular autoregulation, although we found evidence of some fast flow during the onset of sepsis. While no tissue oxygenation data were collected in our model, tissue oxygenation measurements made in the septic heart [33] and simulations of tissue PO_2_ in septic skeletal muscle [34] have suggested the septic tissue is hypoxic, but not anoxic.

### Capillary O_2_ transport—30-second RBC SR–SO_2_–qO_2_ profiles

The imaging technique used in this study acquired high-resolution information on capillary RBC hemodynamics (RBC velocity and lineal density) and RBC hemoglobin O_2_ saturation (SO_2_). From this dynamic information we calculated RBC supply rate (SR) and O_2_ supply rate (qO_2_) in a capillary segment. The technology allowed a direct evaluation of capillary O_2_ transport parameters at locations in the microcirculation where the majority of O_2_ is off-loaded to tissue and RBC hemoglobin O_2_ saturations are at their lowest values. Deviations in the linear relationship between RBC SR and qO_2_ in sepsis animals (Additional file 6) suggested increased heterogeneity in the underlying factors affecting the SR–qO_2_ relationship, including heterogeneous tissue O_2_ consumption, maldistribution of capillary flow and impaired microvascular autoregulation.

The observed altered functional capillary density, increased capillary stopped-flow and capillaries with low O_2_ supply rates in the presence of very fast capillaries with high O_2_ supply rates were indications of increased microvascular heterogeneity, a maldistribution of capillary blood flow and a loss of microvascular autoregulation. Our findings of increased variability in the O_2_ supply rates and delayed capillary responses within hypoxic capillaries suggested the mechanism by which the RBC responds to hypoxic tissue and signals the vasculature to increase flow had been compromised during the onset of sepsis. Theoretically, arrested RBCs in stopped-flow capillaries would have the greatest potential to release ATP in response to hypoxic conditions increasing flow into the affected area. Evidence of increased capillary stopped-flow in sepsis is another indication that autoregulatory mechansims were severely impaired.

### Microvascular autoregulation

While we found evidence of a delayed capillary response within hypoxic capillaries, our finding of decreased RBC O_2_-dependent ATP efflux was initially somewhat surprising given the low RBC SO_2_ observed in some capillaries, as increased O_2_ off-loading should have induced a conformational change in hemoglobin that triggers increased ATP efflux and endothelial signaling. However, we found the opposite as ATP efflux decreased in hypoxic septic RBCs. Consistent with this inhibition of RBC ATP efflux and decreased plasma ATP levels in septic rats is the finding that plasma ATP levels are decreased in critically ill patients [35].

The association of impaired RBC O_2_-dependent ATP efflux with increased plasma NOx and lactate suggested that multiple mechanisms are involved in modulating microvascular autoregulation. In addition to metabolic factors, since erythrocyte deformation induces ATP release [36–39], the possible inhibitory effect of decreased RBC deformability during sepsis [22, 40, 41] on impaired RBC ATP efflux [42] must also be considered. Since we have previously shown that RBC deformability rapidly decreased during the onset of septic injury (by 3–6 hours in this animal model [22]) and decreased RBC deformability inhibits RBC O_2_-dependent ATP release [42], it is possible that changes in the biophysical properties of the RBC membrane may be a mechanism whereby RBC O_2_-dependent ATP efflux was impaired during sepsis. Whether age renders RBCs more susceptible to decreased deformability [41], or a particular subset of RBCs associated with decreased deformability [22] leads to impaired RBC O_2_-dependent ATP efflux is unknown.

In addition to biophysical changes in RBC deformability, biochemical inhibition of RBC glycolysis may be another factor in impaired RBC O_2_-dependent ATP efflux. This is consistent with in vitro experiments reporting that both NO [24] and lactate [43] inhibit RBC O_2_-dependent ATP efflux and the general principle that inhibiting RBC glycolysis impairs RBC O_2_-dependent ATP efflux [13]. As well, peroxynitrite, a derivative of NO and product of the reaction with superoxide anion, has been reported to both stimulate RBC glycolysis at low concentrations via band3 phosphorylation and irreversibly inhibit RBC glycolysis at higher concentrations [44].

In addition to impaired RBC O_2_-dependent ATP signaling, we recognize that impaired electrical coupling of endothelial cell signaling [9] and impaired integrated capillary signaling due to increased capillary stopped-flow [45] at the overall network level of autoregulation may also have been factors in the observed impaired microvascular autoregulation. While it was beyond the scope of this study, we also note that deoxyhemoglobin has been reported to convert nitrite anion to nitric oxide [46], raising the possibility that RBCs within hypoxic capillaries were able to exert a dual level of control over microvascular autoregulation by 1) inhibiting ATP release [24] (the hypoxic ATP signal from the RBC) and/or 2) inhibiting endothelial cell communication via NO release [9] (the relay mechanism by which hypoxic regions communicate with resistance vessels to increase downstream flow).

However, since capillaries are not surrounded by smooth muscle any NO or NO derivatives released from hypoxic RBCs would have no direct vasodilatory effect at the venular ends of skeletal muscle capillary networks, where the lowest RBC O_2_ saturations are detected, and thus neither of the reported hemoglobin-mediated vascular modulators, nitrite [46] nor the more controversial S-nitrosohemoglobin [47–49], were capable of having direct vasodilatory effects in the capillary networks where the lowest RBC hemoglobin O_2_ saturations have been detected. The resistance vessels upstream of the capillary network are surrounded by smooth muscle and are NO targets; however, arterial O_2_ saturations are unchanged in this sepsis model making release of NO from RBCs (or ATP release) along the arterial tree less likely. However, it is conceivable that feeding arterioles neighboring hypoxic tissue regions could be NO targets. Thus the source and targets of NO within the microvascular system during sepsis become of paramount importance in terms of microvascular autoregulation.

While NO is known to inhibit microvascular autoregulation at multiple points in the system (Fig. 5), and may be acting in a negative feedback loop controlling RBC function, we found that increases in arterial NOx correlated with increased capillary RBC supply rate in septic skeletal muscle, suggesting a shift from local control of capillary perfusion via endothelial NOS/NO to a more uncontrolled, but faster delivery of blood flow, as skeletal muscle iNOS/NO rapidly increased in this model [2]. Consistent with this observation, iNOS/NO overproduction is considered a factor for increased coronary circulation during sepsis [50]. Increased NO is also responsible for systemic vasodilation and arteriolar hyporesponsiveness [29, 30]. Thus the pleotropic effects of NO on the cardiovascular system in general and the microcirculation in particular place NO in a central role in modulating microvascular autoregulation. Of further significance to overall organ function during sepsis is that NO inhibits mitochondrial respiration [26, 27] dampening O_2_ consumption during the onset of sepsis in our experimental model [2] and seemingly inhibiting O_2_ consumption when microvascular O_2_ delivery is compromised. Decreasing oxygen consumption in hypoxic regions is possibly an additional protective mechanism [51] that prevents tissue anoxia and certain cell death by decreasing O_2_ consumption and thereby increasing O_2_ diffusion distances in septic tissue with decreased capillary density. As well, similar responses in terms of NO upregulation and microvascular derangements are evident in the septic diaphragm and heart. If impairment of microvascular autoregulation does indeed exist in other septic organs, it may help explain altered gene expression in the septic heart [33], as it responds to local hypoxia. Additional file 7 discusses broader implications of impaired microvascular autoregulation.

### Study limitations and considerations

This study was specifically designed to consider skeletal muscle microvascular function at the capillary level and test the null hypothesis that sepsis has no effect on RBC O_2_-dependent ATP efflux. Changes in capillary O_2_ supply rate are due in part to upstream changes in arteriolar tone distant from sites where RBC O_2_ saturation is lowest (the venular ends of capillary networks) indicating that conducted microvascular responses [16, 25, 45] are integral to microvascular autoregulation. The other important distinctions to be made are: 1) the septic injury in this study does not involve systemic hypoxia, as arterial O_2_ saturations were normal; rather, altered functional capillary density and micro-regions within capillary networks with stopped-flow or decreased capillary O_2_ supply cause local hypoxia and thus a different mechanism is likely involved than that of hypoxic vasodilation [23, 46, 52]; 2) the skeletal muscle NO environment in this model is known to be due to an upregulation of iNOS [2]; 3) microvascular derangements exist in the face of hypotensive [2], “relatively preserved” [7] and even normotensive blood pressure [3, 4] with fluid resuscitation, normal arterial O_2_ concentration and cardiac output [3, 4]. Thus microvascular dysfunction is apparently independent of mean arterial pressure and may be masked by seemingly normal cardiovascular parameters.

Increased arterial and tissue NOx previously reported in this sepsis model [2] are suspected to result from NO oxidation reactions and the scavenging effects of oxy- and deoxyhemoglobin on NO [53, 54]; however, previous EPR (Electron paramagnetic resonance) spectroscopy studies in our model have shown an accumulation of hemoglobin-NO [22] in the septic RBC suggesting that NO could be accumulating within the RBC or regenerated by the RBC itself [46], although the extent and effect of such a reaction in the context of tissue iNOS/NO upregulation and overproduction [2] is unclear. While NO generated within the RBC, possibly by an RBC NOS [55], could inhibit RBC glycolysis [44] effectively reducing the RBC O_2_-dependent ATP efflux [24] in a negative feedback manner, the mechanism in sepsis is not understood. Any possible effects of NO_2_^−^ potentiating ATP efflux [56] are unknown.

### Future work

This study raises an important question—specifically, can the septic microcirculation be rescued by preventing the delayed capillary response within hypoxic capillaries with low RBC SO_2_ or rescuing RBC O_2_-dependent ATP signaling? Or is it more important to consider the entire microvascular autoregulatory system as a functional unit [1], including RBC O_2_-dependent ATP signaling, endothelial cell communication, vascular reactivity and NO overproduction that together need to be regulated and restored in order to rescue the septic microcirculation and improve capillary response times.

## Conclusion

While septic erythrocytes remained capable of off-loading increased amounts of O_2_ within septic capillaries, both the capillary response within hypoxic capillaries and the septic RBC O_2_-dependent ATP response to hypoxia were impaired. This impairment of the RBC to fully respond to its O_2_ environment was likely a factor in the delayed capillary response with low RBC O_2_ saturations, although other factors were likely involved including attenuated endothelial cell-conducted vascular response and altered vasoreactivity. Accordingly, treatments aimed at restoring the autoregulation of the septic microcirculation may be of benefit to the septic patient, provided the complete microvascular autoregulatory system can be rescued simultaneously. However, further research will be required to form a more complete understanding of how microvascular autoregulation is operating in both health and disease states.

## Key messages

- Sepsis attenuates the capillary response within hypoxic capillaries.
- Septic erythrocytes are impaired from releasing ATP in response to hypoxic conditions.
- Sepsis-induced impairment of microvascular autoregulation is partially off-set by increased capillary RBC supply rate, which correlates with increased plasma NOx.
- Sepsis induces profound disturbances in microvascular function and control.
- Microvascular autoregulation is impaired at three levels: 1) the RBC level, 2) the capillary level and 3) the overall capillary network level.

## Additional files

Additional file 1:
**Data supplement including information on methods.** (PDF 927 kb) Additional file 2:
**Video clip of skeletal muscle capillary red blood cell (RBC) hemodynamics.** (MOV 4983 kb) Additional file 3:
**Video clip of skeletal muscle capillary red blood cell hemoglobin O**_**2**_ **saturation (RBC SO**_**2**_ **).** (MOV 4923 kb) Additional file 4:
**RBC supply rate (SR) and O**_**2**_ **supply rate (qO**_**2**_ **) heterogeneity.** (PDF 285 kb) Additional file 5:
**Control capillary RBC supply rate and O**_**2**_ **supply rate 95 % confidence intervals.** (PDF 56 kb) Additional file 6:
**Relationship between capillary RBC supply rate and O**_**2**_ **supply rate.** (PDF 234 kb) Additional file 7:
**Broader implications of impaired microvascular autoregulation in skeletal muscle.** (PDF 50 kb)

## Abbreviations

CLP: Cecal ligation and perforation
iNOS: Inducible nitric oxide synthase
NO: Nitric oxide
NOx: NO_2_^−^ + NO_3_^−^
O_2_: Oxygen
OD: Optical density
P2Y: Purinergic type 2
PO_2_: Partial pressure of oxygen
qO_2_: Capillary oxygen supply rate
RBC: Red blood cell
SO_2_: Oxygen saturation
SR: RBC supply rate

## References

[1] Bateman RM, Sharpe MD, Ellis CG (2003). Bench-to-bedside review: microvascular dysfunction in sepsis—hemodynamics, oxygen transport, and nitric oxide. Crit Care..

[2] Bateman RM, Sharpe MD, Goldman D, Lidington D, Ellis CG (2008). Inhibiting nitric oxide overproduction during hypotensive sepsis increases local oxygen consumption in rat skeletal muscle. Crit Care Med..

[3] Ellis CG, Bateman RM, Sharpe MD, Sibbald WJ, Gill R (2002). Effect of a maldistribution of microvascular blood flow on capillary O(2) extraction in sepsis. Am J Physiol Heart Circ Physiol..

[4] Lam C, Tyml K, Martin C, Sibbald W (1994). Microvascular perfusion is impaired in a rat model of normotensive sepsis. J Clin Invest..

[5] De Backer D, Creteur J, Preiser JC, Dubois MJ, Vincent JL (2002). Microvascular blood flow is altered in patients with sepsis. Am J Respir Crit Care Med..

[6] Hernandez G, Boerma EC, Dubin A, Bruhn A, Koopmans M, Edul VK (2013). Severe abnormalities in microvascular perfused vessel density are associated to organ dysfunctions and mortality and can be predicted by hyperlactatemia and norepinephrine requirements in septic shock patients. J Crit Care.

[7] De Backer D, Donadello K, Sakr Y, Ospina-Tascon G, Salgado D, Scolletta S (2013). Microcirculatory alterations in patients with severe sepsis: impact of time of assessment and relationship with outcome. Crit Care Med..

[8] Trzeciak S, Dellinger RP, Parrillo JE, Guglielmi M, Bajaj J, Abate NL (2007). Early microcirculatory perfusion derangements in patients with severe sepsis and septic shock: relationship to hemodynamics, oxygen transport, and survival. Ann Emerg Med.

[9] McKinnon RL, Lidington D, Bolon M, Ouellette Y, Kidder GM, Tyml K (2006). Reduced arteriolar conducted vasoconstriction in septic mouse cremaster muscle is mediated by nNOS-derived NO. Cardiovasc Res..

[10] Ellsworth ML, Forrester T, Ellis CG, Dietrich HH (1995). The erythrocyte as a regulator of vascular tone. Am J Physiol..

[11] Sprague RS, Hanson MS, Achilleus D, Bowles EA, Stephenson AH, Sridharan M (2009). Rabbit erythrocytes release ATP and dilate skeletal muscle arterioles in the presence of reduced oxygen tension. Pharmacol Rep..

[12] Bergfeld GR, Forrester T (1992). Release of ATP from human erythrocytes in response to a brief period of hypoxia and hypercapnia. Cardiovasc Res..

[13] Jagger JE, Bateman RM, Ellsworth ML, Ellis CG (2001). Role of erythrocyte in regulating local O2 delivery mediated by hemoglobin oxygenation. Am J Physiol Heart Circ Physiol..

[14] Lewis IA, Campanella ME, Markley JL, Low PS (2009). Role of band 3 in regulating metabolic flux of red blood cells. Proc Natl Acad Sci U S A..

[15] Messana I, Orlando M, Cassiano L, Pennacchietti L, Zuppi C, Castagnola M (1996). Human erythrocyte metabolism is modulated by the O2-linked transition of hemoglobin. FEBS Lett..

[16] Collins DM, McCullough WT, Ellsworth ML (1998). Conducted vascular responses: communication across the capillary bed. Microvasc Res..

[17] Ellsworth ML (2000). The red blood cell as an oxygen sensor: what is the evidence?. Acta Physiol Scand..

[18] Bateman RM, Ellis CG, Sharpe MD, Mehta S, Freeman DJ (2001). Effect of hemolyzed plasma on the batch measurement of nitrate by nitric oxide chemiluminescence. Clin Chem..

[19] Bateman RM, Ellis CG, Freeman DJ (2002). Optimization of nitric oxide chemiluminescence operating conditions for measurement of plasma nitrite and nitrate. Clin Chem..

[20] Japee SA, Pittman RN, Ellis CG (2005). A new video image analysis system to study red blood cell dynamics and oxygenation in capillary networks. Microcirculation..

[21] Ellsworth ML, Pittman RN, Ellis CG (1987). Measurement of hemoglobin oxygen saturation in capillaries. Am J Physiol..

[22] Bateman RM, Jagger JE, Sharpe MD, Ellsworth ML, Mehta S, Ellis CG (2001). Erythrocyte deformability is a nitric oxide-mediated factor in decreased capillary density during sepsis. Am J Physiol Heart Circ Physiol..

[23] Crawford JH, Isbell TS, Huang Z, Shiva S, Chacko BK, Schechter AN (2006). Hypoxia, red blood cells, and nitrite regulate NO-dependent hypoxic vasodilation. Blood..

[24] Olearczyk JJ, Ellsworth ML, Stephenson AH, Lonigro AJ, Sprague RS (2004). Nitric oxide inhibits ATP release from erythrocytes. J Pharmacol Exp Ther..

[25] McKinnon RL, Bolon ML, Wang HX, Swarbreck S, Kidder GM, Simon AM (2009). Reduction of electrical coupling between microvascular endothelial cells by NO depends on connexin37. Am J Physiol Heart Circ Physiol..

[26] Davies NA, Cooper CE, Stidwill R, Singer M (2003). Inhibition of mitochondrial respiration during early stage sepsis. Adv Exp Med Biol..

[27] Frost MT, Wang Q, Moncada S, Singer M (2005). Hypoxia accelerates nitric oxide-dependent inhibition of mitochondrial complex I in activated macrophages. Am J Physiol Regul Integr Comp Physiol..

[28] Tyml K, Yu J, McCormack DG (1998). Capillary and arteriolar responses to local vasodilators are impaired in a rat model of sepsis. J Appl Physiol..

[29] Hollenberg SM, Cunnion RE, Zimmerberg J (1993). Nitric oxide synthase inhibition reverses arteriolar hyporesponsiveness to catecholamines in septic rats. Am J Physiol..

[30] Hollenberg SM, Piotrowski MJ, Parrillo JE (1997). Nitric oxide synthase inhibition reverses arteriolar hyporesponsiveness to endothelin-1 in septic rats. Am J Physiol..

[31] Vary TC (1996). Sepsis-induced alterations in pyruvate dehydrogenase complex activity in rat skeletal muscle: effects on plasma lactate. Shock..

[32] Scott JA, Mehta S, Duggan M, Bihari A, McCormack DG (2002). Functional inhibition of constitutive nitric oxide synthase in a rat model of sepsis. Am J Respir Crit Care Med..

[33] Bateman RM, Tokunaga C, Kareco T, Dorscheid DR, Walley KR (2007). Myocardial hypoxia-inducible HIF-1alpha, VEGF, and GLUT1 gene expression is associated with microvascular and ICAM-1 heterogeneity during endotoxemia. Am J Physiol Heart Circ Physiol..

[34] Goldman D, Bateman RM, Ellis CG (2004). Effect of sepsis on skeletal muscle oxygen consumption and tissue oxygenation: interpreting capillary oxygen transport data using a mathematical model. Am J Physiol Heart Circ Physiol..

[35] Chida J, Ono R, Yamane K, Hiyoshi M, Nishimura M, Onodera M (2013). Blood lactate/ATP ratio, as an alarm index and real-time biomarker in critical illness. PLoS One..

[36] Faris A, Spence DM (2008). Measuring the simultaneous effects of hypoxia and deformation on ATP release from erythrocytes. Analyst..

[37] Forsyth AM, Wan J, Owrutsky PD, Abkarian M, Stone HA (2011). Multiscale approach to link red blood cell dynamics, shear viscosity, and ATP release. Proc Natl Acad Sci U S A..

[38] Sprague RS, Ellsworth ML, Stephenson AH, Kleinhenz ME, Lonigro AJ (1998). Deformation-induced ATP release from red blood cells requires CFTR activity. Am J Physiol..

[39] Sprague RS, Ellsworth ML, Stephenson AH, Lonigro AJ (1996). ATP: the red blood cell link to NO and local control of the pulmonary circulation. Am J Physiol..

[40] Baskurt OK, Gelmont D, Meiselman HJ (1998). Red blood cell deformability in sepsis. Am J Respir Crit Care Med..

[41] Condon MR, Kim JE, Deitch EA, Machiedo GW, Spolarics Z (2003). Appearance of an erythrocyte population with decreased deformability and hemoglobin content following sepsis. Am J Physiol Heart Circ Physiol..

[42] Sridharan M, Sprague RS, Adderley SP, Bowles EA, Ellsworth ML, Stephenson AH (2010). Diamide decreases deformability of rabbit erythrocytes and attenuates low oxygen tension-induced ATP release. Exp Biol Med..

[43] Rozier MD, Zata VJ, Ellsworth ML (2007). Lactate interferes with ATP release from red blood cells. Am J Physiol Heart Circ Physiol..

[44] Mallozzi C, Di Stasi AM, Minetti M (1997). Peroxynitrite modulates tyrosine-dependent signal transduction pathway of human erythrocyte band 3. FASEB J..

[45] Ellis CG, Milkovich S, Goldman D (2012). What is the efficiency of ATP signaling from erythrocytes to regulate distribution of O(2) supply within the microvasculature?. Microcirculation..

[46] Cosby K, Partovi KS, Crawford JH, Patel RP, Reiter CD, Martyr S (2003). Nitrite reduction to nitric oxide by deoxyhemoglobin vasodilates the human circulation. Nat Med..

[47] Isbell TS, Sun CW, Wu LC, Teng X, Vitturi DA, Branch BG (2008). SNO-hemoglobin is not essential for red blood cell-dependent hypoxic vasodilation. Nat Med..

[48] Zhang R, Hess DT, Qian Z, Hausladen A, Fonseca F, Chaube R (2015). Hemoglobin betaCys93 is essential for cardiovascular function and integrated response to hypoxia. Proc Natl Acad Sci U S A..

[49] Liu Y, Sun CW, Honavar J, Townes T, Patel RP (2013). Role of the b93cys, ATP and adenosine in red cell dependent hypoxic vasorelaxation. Int J Physiol Pathophysiol Pharmacol..

[50] Rudiger A, Singer M (2007). Mechanisms of sepsis-induced cardiac dysfunction. Crit Care Med..

[51] Singer M, De Santis V, Vitale D, Jeffcoate W (2004). Multiorgan failure is an adaptive, endocrine-mediated, metabolic response to overwhelming systemic inflammation. Lancet..

[52] Umbrello M, Dyson A, Pinto BB, Fernandez BO, Simon V, Feelisch M (2014). Short-term hypoxic vasodilation in vivo is mediated by bioactive nitric oxide metabolites, rather than free nitric oxide derived from haemoglobin-mediated nitrite reduction. J Physiol..

[53] Isbell TS, Gladwin MT, Patel RP (2007). Hemoglobin oxygen fractional saturation regulates nitrite-dependent vasodilation of aortic ring bioassays. Am J Physiol Heart Circ Physiol..

[54] Azarov I, Huang KT, Basu S, Gladwin MT, Hogg N, Kim-Shapiro DB (2005). Nitric oxide scavenging by red blood cells as a function of hematocrit and oxygenation. J Biol Chem..

[55] Ulker P, Gunduz F, Meiselman HJ, Baskurt OK (2013). Nitric oxide generated by red blood cells following exposure to shear stress dilates isolated small mesenteric arteries under hypoxic conditions. Clin Hemorheol Microcirc..

[56] Cao Z, Bell JB, Mohanty JG, Nagababu E, Rifkind JM (2009). Nitrite enhances RBC hypoxic ATP synthesis and the release of ATP into the vasculature: a new mechanism for nitrite-induced vasodilation. Am J Physiol Heart Circ Physiol..

[57] Campanella ME, Chu H, Low PS (2005). Assembly and regulation of a glycolytic enzyme complex on the human erythrocyte membrane. Proc Natl Acad Sci U S A..

[58] Sega MF, Chu H, Christian J, Low PS (2012). Interaction of deoxyhemoglobin with the cytoplasmic domain of murine erythrocyte band 3. Biochemistry..

[59] Pawloski JR, Hess DT, Stamler JS (2001). Export by red blood cells of nitric oxide bioactivity. Nature..

