# Supplementary material for: Sepsis impairs microvascular autoregulation and delays capillary response within hypoxic capillaries
Source: Crit Care. 2015 Nov 5;19:389. doi: 10.1186/s13054-015-1102-7 (PMC4634189; doi:10.1186/s13054-015-1102-7)
Supplement: Additional file 1: — Data supplement including information on methods. (PDF 927 kb) [file 13054_2015_1102_MOESM1_ESM.pdf]

Sepsis impairs microvascular autoregulation and delays capillary response within hypoxic capillaries

Ryon M Bateman, Michael D Sharpe, Justin E Jagger and Christopher G Ellis

Data supplement

Animal model of sepsis

Sepsis was studied using an established acute fecal peritonitis rat model [1, 2]. Experimental and surgical protocols were approved by the University of Western Ontario Council on Animal Care. Animal surgery and muscle preparation were performed in two stages. In the first stage, male Sprague-Dawley rats, which had been acclimated to the animal facility for several weeks, weighing between 160 and 170 grams, were transferred from the animal facility to the surgical/imaging area up to an hour before surgery. They had free access to rat chow and water to this point. Surgery started between 9 and 10am. Animals were randomized to sham (sham, n=6) and cecal ligation and perforation (CLP, n=5) procedures.

A systemic inflammatory response secondary to fecal peritonitis was initiated by expressing the fecal contents of an animal into its peritoneal cavity. In brief, a midline incision was made in the anesthetized animal and its cecum exteriorized. The cecum was then devascularized and ligated distal to its ileocecal valve. Using a cauterizer, a small 5 mm incision was made in the cecum and it was

returned to the peritoneal cavity. The fecal contents were then expressed into the peritoneal cavity and the incision closed in two layers. The animal was then rotated 180 degrees on the surgical table. This was time zero of the experiment. Next, a small incision was made in the right carotid artery and polyethylene-10 (Clay Adams) tubing, attached to a blood pressure analyzer (Digi-Med Blood Pressure Analyzer, Micro-Med, Louisville, KY), was advanced into the vessel. The left jugular vein was then cannulated for continuous infusion of anesthesia (sodium pentobarbital (18 $mg \cdot kg^{-1} \cdot hr^{-1}$) and fluid resuscitation (0.9% saline, 10 $ml.kg^{-1}.hr^{-1}$), by advancing Bio-Sil tubing (Silmed, Akron, OH) attached to an infusion pump into the vessel. Following cannulation, the animal underwent a rapid tracheostomy and was put on a ventilator (30%:70% O_2/N_2, respiratory rate 72-85 breaths/min and tidal volume 1.6-2.2 mL – Harvard Rodent Ventilator, Diversified Equipment, Lorton, VA). Inspired oxygen was monitored by an inline O_2 analyzer (Critikon,Oxychek). Ventilation was adjusted to bring blood gases into the normal range (PO_2: 85-100 mmHg, PCO_2: 35-45, pH: 7.3-7.4) and not adjusted for the remainder of the investigation. The surgical time for the first stage was between 45 and 60 minutes.

The second stage of the surgical procedure was the preparation of the right hind limb *extensor digitorum longus* (EDL) skeletal muscle, which was used for microvascular imaging. An incision was made along the overlying skin and fascia to expose the relatively thin EDL muscle. It was then blunt dissected from

surrounding muscles and a suture tied to its tendon. After severing the tendon, the free EDL was moistened with warm saline and covered with a piece of saran. The animal was then repositioned on the imaging stage in the recovery position. The EDL was reflected away from the leg, perpendicular to the body, taking care not to twist the muscle and positioned in the optical path of the imaging system. It was held at *in situ* length by fastening the suture to the stage. The muscle was remoistened with warm saline, covered with fresh Saran Wrap and a glass cover slip, and allowed to stabilize for at least 1 hour. The experiment was terminated if any muscle damage was detected. The surgical time for the muscle preparation was approximately 1 hour. Animal core temperature was maintained (36.5-37.2 ^{o}C) by heat lamp and monitored by a rectal temperature probe.

Blood samples and lactate analysis

During the surgical phase of the experiment, arterial blood samples were obtained for blood gas analysis (ABL Radiometer Blood Gas Analyzer, ABL500, Copenhagen, Denmark). At the end of each 6 hour experiment, heparinized arterial blood samples were collected for blood gas analysis, lactate analysis (Synchron System, Beckman Coulter, Ca) and for RBC ATP efflux measurements.

RBC ATP measurement

At the 6-hr end point of the experiment, whole blood was collected in a heparinized tube and kept on ice. Blood was mixed gently and introduced into a

humidified gas exchange chamber (fitted with 1meter long 0.1 mm inner diameter silastic tubing pretreated with heparinized saline) held at 37C, as previously described [3]. Erythrocytes were then equilibrated to normoxic conditions (N) (21% O_2, 5% CO_2, balance N2 for 5 minutes). 10ul of treated blood was then diluted into 10 ml of physiological buffer (pH 7.4, 300 mosM, 37C, consisting of 140.5 mM NaCl, 4.7mM KCl, 2.0 mM CaCl2, 1.2 mM MgSO4, 21.0 mM tris(hydroxymethyl)aminoethane with 1 g/L glucose and 5 g/l albumin) and injected into a custom designed photon counting chamber. In brief, the equilibrated blood samples were injected into a light tight box containing a photocuvette preloaded with a luciferin-luciferase cocktail (250ul firefly extract (FLE-50, Sigma, St.Louis, MO) and 250ul synthetic D-luciferin (50 mg/100ml distilled water (Sigma, St.Louis, MO)). The light emitted from the ATP luciferin-luciferase reaction was detected by a photomultiplier tube (model HC135-01, Hamamatsu, Japan) and the resulting signal pulse sent to a personal computer for analysis using custom designed software. The sample was then exposed to hypoxic conditions (H) (0% O_2, 5% CO_2, balance N2 for 5 minutes) and ATP analysis repeated. Since only extracellular ATP was measured in the assay, it was not necessary to separate the supernatant. All samples were tested for the presence of hemolysis and rejected if hemoglobin was detected in the supernatant. All samples were run in triplicate. Analysis was calibrated against known concentrations of ATP.

Functional microvascular imaging

A custom designed dual wavelength imaging system was used to visualize and record real-time video images [4-6] of the rat EDL skeletal muscle microcirculation under both sham and septic conditions. The EDL muscle was illuminated by a 100W xenon light source mounted on a Diaphot 300 inverted microscope with a 20x/0.4 NA objective (Nikon, Yokohama, Japan). Transmitted light was directed to a beam splitter fitted with 420 and 430nm interference filters. Optical density information from the skeletal muscle field of view, at 420 and 430 nm, was captured by two CCD cameras (MTI CCDC72, North Reading, MA) and recorded on two sets of S-VHS video tapes (video monitor, model WV-5410 and video cassette recorder, model AG-7300, Panasonic), one for each wavelength, respectively.

Off-line image analysis was performed on a Silicon Graphics workstation (St-Laurent, QU). 30-second video sequences of capillaries of interest were captured using custom computer software and stored on hard disc as two sets of 900 image files in TIFF format. Optical density (OD) information was calculated from OD = log(Io/I), where Io is the incident light intensity and I is the transmitted light passing through the RBC, and used to calculate capillary geometry (vessel diameter and length), RBC hemodynamics (RBC velocity (um/s), lineal density (RBC/mm), supply rate (RBC/s)) and individual RBC oxygen saturation (SO_2). Variation in light intensity (420 nm) as RBCs passed through capillaries was used

to generate an image of the flowing RBC column and vessel diameter, and used to calculate vessel geometry [5]. RBC velocity was computed using frame-by-frame spatial correlation and RBC lineal density was computed by determining the number of RBCs in a given capillary segment in each video frame [6]. The RBC supply rate was then calculated as the product of RBC velocity and lineal density [5]. RBC SO_2 (RBC hemogloblin oxygen saturation (%)) was calculated from the 430/420-nm OD ratio as previously described [7], based on the equation SO2 = a + b*OD430/OD420, where a and b were determined by *in vivo* calibration against 0 and 100% oxygen. Figure S1, panels A-D, show an example of 30-second RBC hemodynamic and oxygen saturation (SO_2) profiles from a single capillary. Figure S2 and matching video clips show real-time capillary hemodynamics [see Additional File 2] and RBC oxygen saturations [see Additional File 3]. Capillary oxygen supply rate was calculated as: $qO_2 = k*SR*SO_2$, where k is O_2 carrying capacity of RBC at 100% saturation (0.0362 pLO_2/RBC) [4].

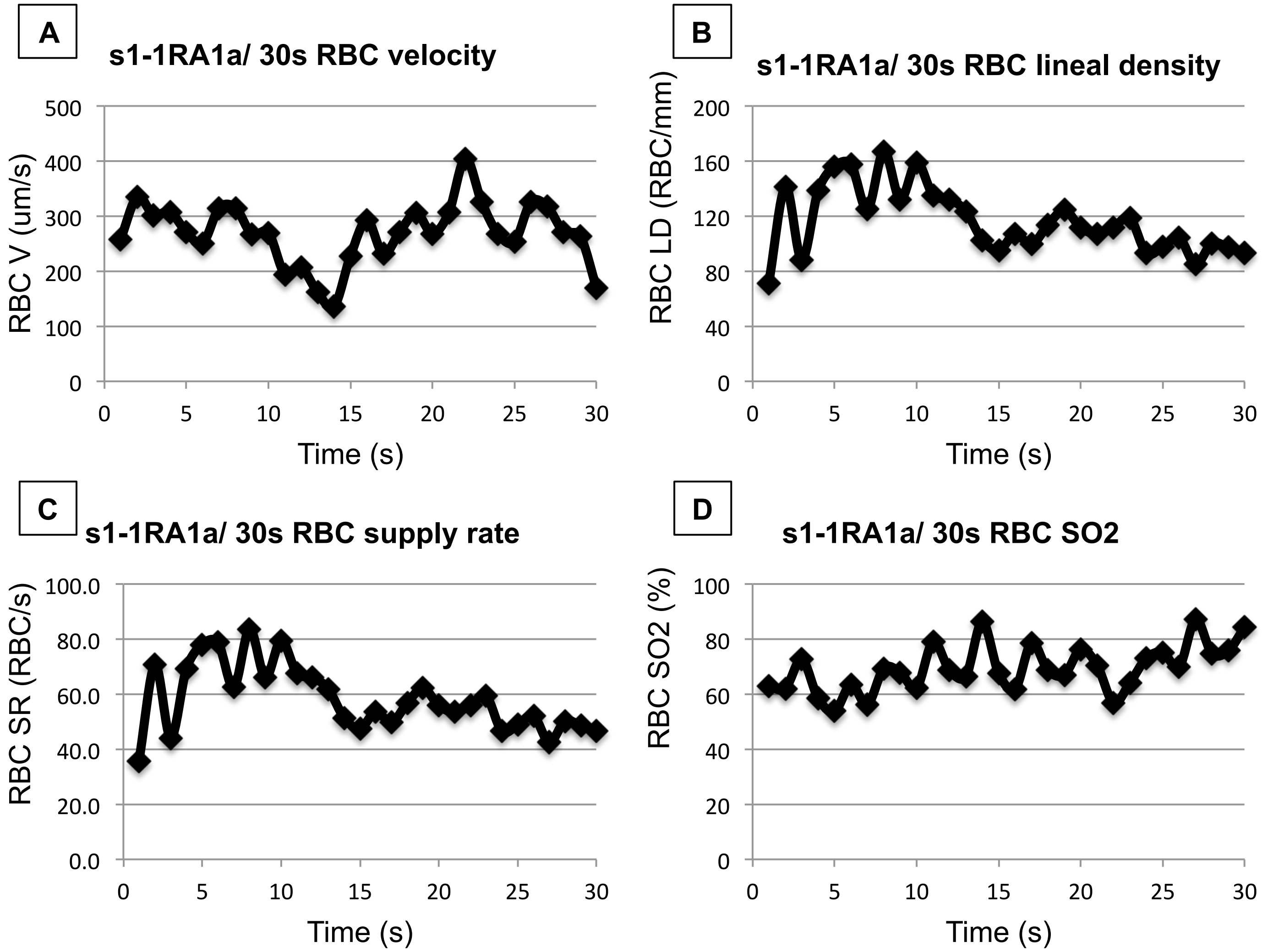

Figure S1. Example of 30-second capillary RBC hemodynamic and RBC oxygen saturation profiles. Panels A-D show examples of 30-second RBC velocity (V(um/s)), lineal density (LD(RBC/mm)) and supply rate (SR(RBC/s)) and RBC oxygen saturation (SO_2(%)) acquired from a single rat EDL skeletal muscle capillary using a dual wavelength imaging system. Note SR = V * LD.

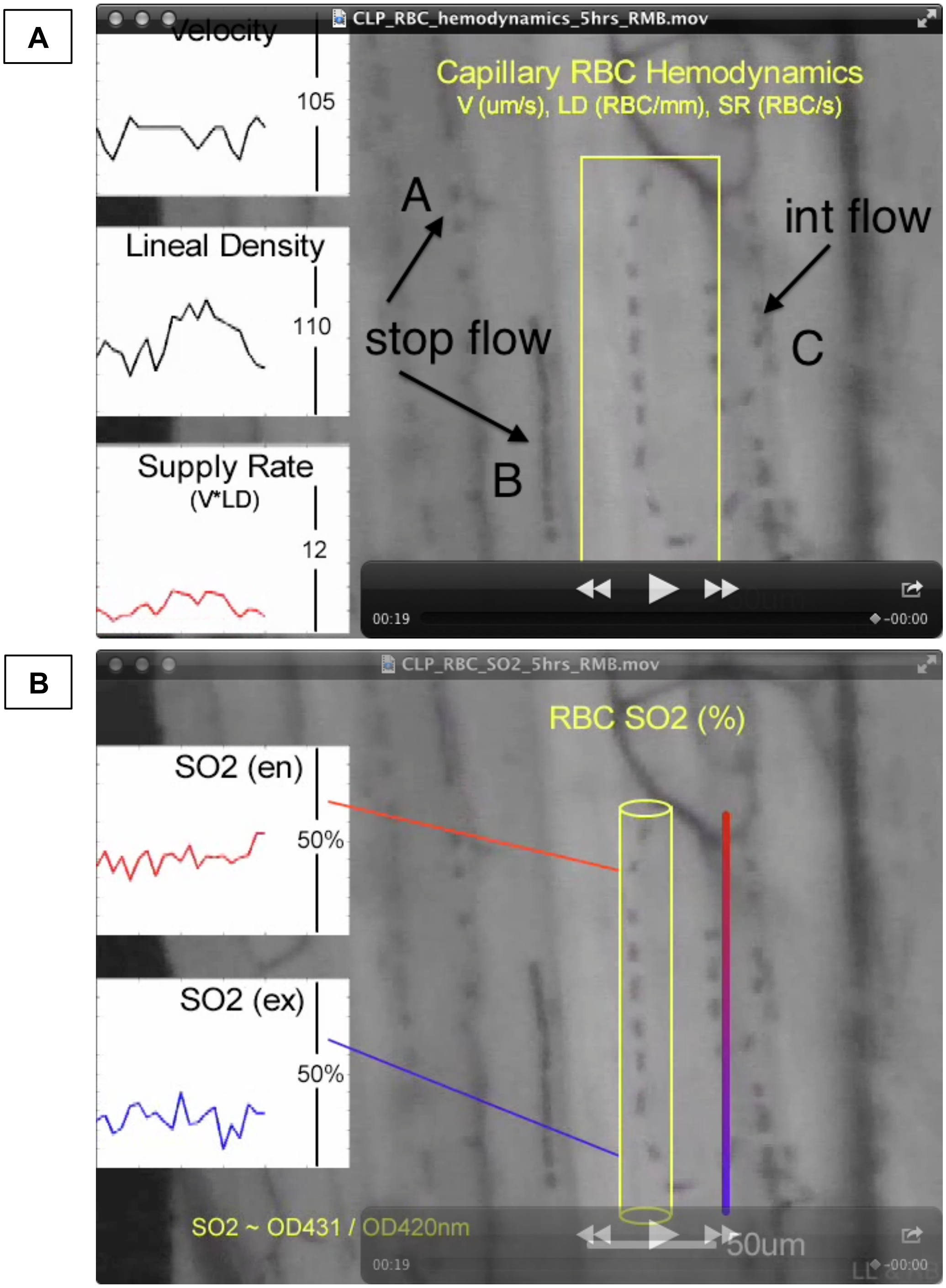

Figure S2. Rat hind limb EDL skeletal muscle microcirculation 5 hours after sepsis. Panel A and video clip 1 show the video image/clip of RBC hemodynamics from a continuously flowing capillary from the EDL skeletal muscle of a septic animal. RBCs are flowing from top to bottom. The left inserts (top to bottom) show the real-time RBC velocity (top), RBC lineal density (middle) and RBC supply rate (bottom), respectively. Note that to the left of the continuous capillary in the marked rectangle some capillaries have stopped flowing (A,B) and to the right a capillary is exhibiting intermittent flow (C). Figure E2, panel B and video clip 2 show the video image/clip of capillary RBC oxygen saturation (SO_2) at the capillary entrance (top) and capillary exit (bottom), respectively.

REFERENCES

1. Bateman RM, Jagger JE, Sharpe MD, Ellsworth ML, Mehta S, Ellis CG: **Erythrocyte deformability is a nitric oxide-mediated factor in decreased capillary density during sepsis.** *American journal of physiology Heart and circulatory physiology* 2001, **280**(6):H2848-2856.
2. Bateman RM, Sharpe MD, Goldman D, Lidington D, Ellis CG: **Inhibiting nitric oxide overproduction during hypotensive sepsis increases local oxygen consumption in rat skeletal muscle**. *Critical care medicine* 2008, **36**(1):225-231.
3. Jagger JE, Bateman RM, Ellsworth ML, Ellis CG: **Role of erythrocyte in regulating local O2 delivery mediated by hemoglobin oxygenation**. *American journal of physiology Heart and circulatory physiology* 2001, **280**(6):H2833-2839.
4. Ellis CG, Bateman RM, Sharpe MD, Sibbald WJ, Gill R: **Effect of a maldistribution of microvascular blood flow on capillary O(2) extraction in sepsis**. *American journal of physiology Heart and circulatory physiology* 2002, **282**(1):H156-164.
5. Japee SA, Pittman RN, Ellis CG: **Automated method for tracking individual red blood cells within capillaries to compute velocity and oxygen saturation**. *Microcirculation* 2005, **12**(6):507-515.

6. Japee SA, Pittman RN, Ellis CG: **A new video image analysis system to study red blood cell dynamics and oxygenation in capillary networks**. *Microcirculation* 2005, **12**(6):489-506.
7. Ellsworth ML, Pittman RN, Ellis CG: **Measurement of hemoglobin oxygen saturation in capillaries**. *The American journal of physiology* 1987, **252**(5 Pt 2):H1031-1040.
