# Supplementary material for: Sepsis impairs microvascular autoregulation and delays capillary response within hypoxic capillaries
Source: Crit Care. 2015 Nov 5;19:389. doi: 10.1186/s13054-015-1102-7 (PMC4634189; doi:10.1186/s13054-015-1102-7)
Supplement: Additional file 4: — RBC supply rate (SR) and O 2 supply rate (qO 2 ) heterogeneity. (PDF 285 kb) [file 13054_2015_1102_MOESM4_ESM.pdf]

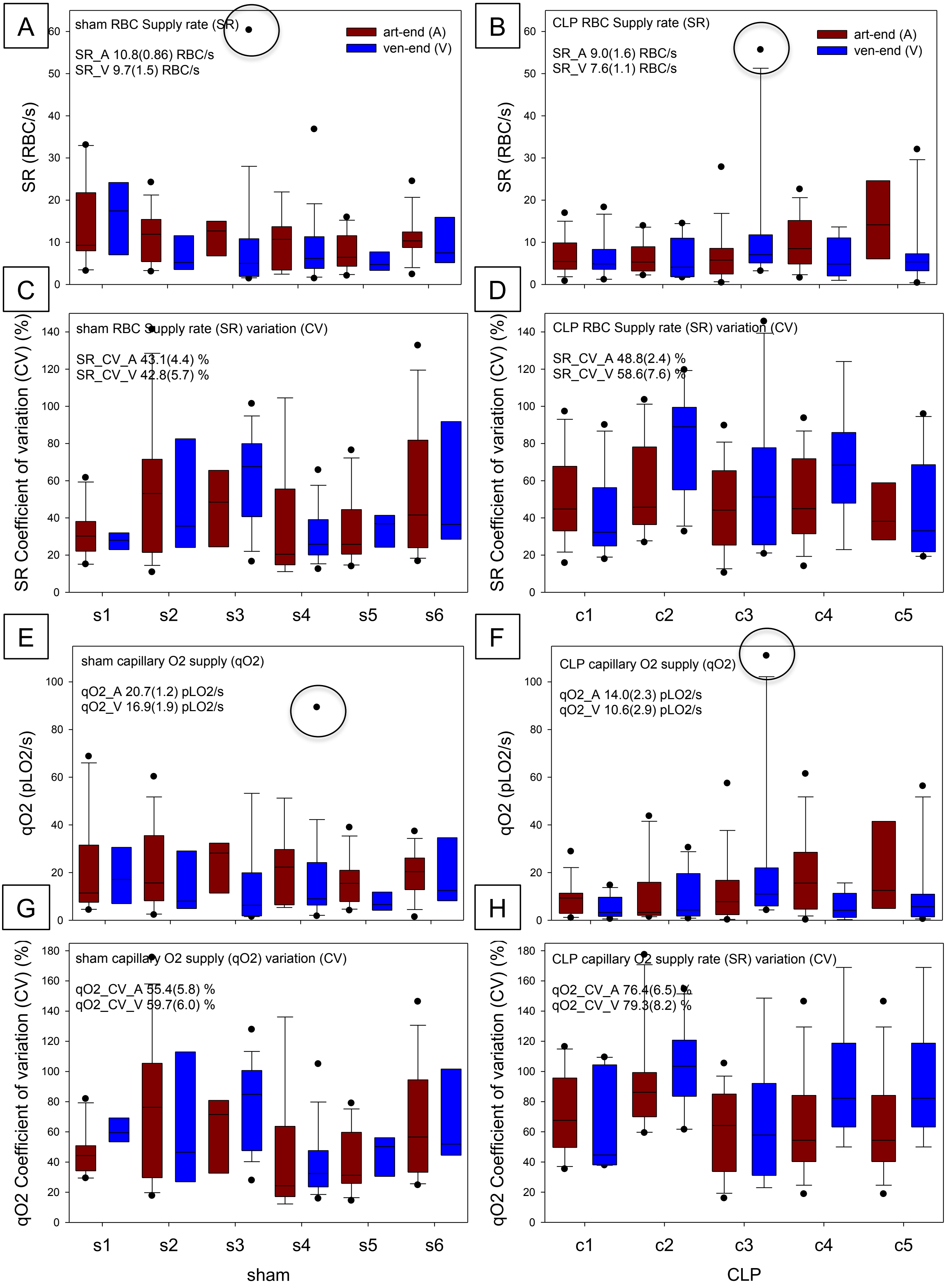
A
sham RBC Supply rate (SR)
art-end (A)
ven-end (V)
SR_A 10.8(0.86) RBC/s
SR_V 9.7(1.5) RBC/s
SR (RBC/s)
B
CLP RBC Supply rate (SR)
SR_A 9.0(1.6) RBC/s
SR_V 7.6(1.1) RBC/s
C
sham RBC Supply rate (SR) variation (CV)
SR_CV_A 43.1(4.4) %
SR_CV_V 42.8(5.7) %
SR Coefficient of variation (CV) (%)
D
CLP RBC Supply rate (SR) variation (CV)
SR_CV_A 48.8(2.4) %
SR_CV_V 58.6(7.6) %
s1 s2 s3 s4 s5 s6
c1 c2 c3 c4 c5
E
sham capillary O2 supply (qO2)
qO2_A 20.7(1.2) pLO2/s
qO2_V 16.9(1.9) pLO2/s
qO2 (pLO2/s)
F
CLP capillary O2 supply (qO2)
qO2_A 14.0(2.3) pLO2/s
qO2_V 10.6(2.9) pLO2/s
G
sham capillary O2 supply (qO2) variation (CV)
qO2_CV_A 55.4(5.8) %
qO2_CV_V 59.7(6.0) %
qO2 Coefficient of variation (CV) (%)
H
CLP capillary O2 supply rate (SR) variation (CV)
qO2_CV_A 76.4(6.5) %
qO2_CV_V 79.3(8.2) %
sham
CLP

Figure S3. RBC supply rate (SR) and capillary oxygen supply (qO2) heterogeneity.

Sham RBC supply rates (SR) and capillary oxygen supply rates (qO_2) and the respective variations (heterogeneity) in their 30-second profiles, are shown in column 1, panels A,C,E,G. Comparable CLP results are shown in column 2, panels B,D,F,H. Heterogeneity was calculated as the 30-second coefficient of variation (CV). Results are summarized in table 2. Note that extremely fast RBC SR and high capillary qO_2 were detected in some experiments (see circled data points).
