# Supplementary material for: Sepsis impairs microvascular autoregulation and delays capillary response within hypoxic capillaries
Source: Crit Care. 2015 Nov 5;19:389. doi: 10.1186/s13054-015-1102-7 (PMC4634189; doi:10.1186/s13054-015-1102-7)
Supplement: Additional file 5: — Control capillary RBC supply rate and O 2 supply rate 95 % confidence intervals. (PDF 56 kb) [file 13054_2015_1102_MOESM5_ESM.pdf]

Table S1. Control capillary RBC SR and qO_2 95% confidence intervals

	Art-end 95% CI	Ven-end 95% CI
SR (RBC/s)	6.2 – 15.0	3.9 – 16.3
qO_2 (pLO_2/s)	10.3 – 30.4	3.3 – 33.5

SR = capillary RBC supply rate. qO_2 = capillary oxygen supply rate. Art-end = capillary arteriolar end. Ven-end = capillary venular-end. 95% CI = 95% confidence interval. Relative to these confidence limits, RBC supply rates (were categorized as either slow (below), average (within) or fast (above-95% CI); while, oxygen supply rates were categorized as either low (below), average (within) or high (above-95% CI).
