# Supplementary material for: Sepsis impairs microvascular autoregulation and delays capillary response within hypoxic capillaries
Source: Crit Care. 2015 Nov 5;19:389. doi: 10.1186/s13054-015-1102-7 (PMC4634189; doi:10.1186/s13054-015-1102-7)
Supplement: Additional file 6: — Relationship between capillary RBC supply rate and O 2 supply rate. (PDF 234 kb) [file 13054_2015_1102_MOESM6_ESM.pdf]

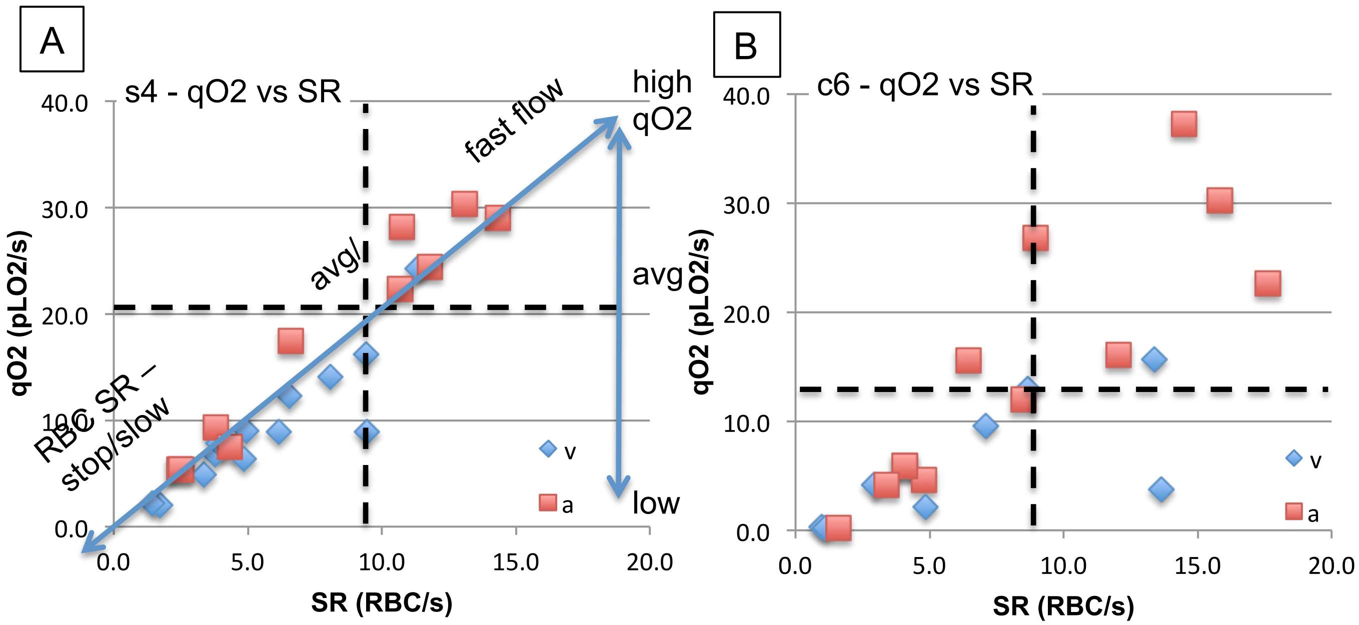

Figure S4 – Relationship between capillary RBC supply rate and oxygen supply rate. Capillary RBC supply rate (SR) and oxygen supply rate (pLO_2/s) measurements were made using a functional microvascular imaging system, as described in methods. Panels A,B show qO_2 vs SR plots for a sham and CLP experiment, respectively. 95% confidence intervals (Table S1) at the art-end (a) and ven-end (v) of capillary networks were used to categorize RBC SR as slow, average or fast and capillary qO_2 as low, average or high. The diagonal line in panel A shows how coupled variables qO_2 and SR follow a straight line in the control animal. The dashed lines show the average qO_2 and SR in each animal. v = venular-end, a = arteriolar-end of capillary segment. Note that since qO_2 and SR variables are coupled ($qO_2 = SR*SO_2*k$), data points are expected to fall on a straight line. Marked along the diagonal blue line, in panel A, are the relative slow, average and fast SR categories as determined by the 95% confidence intervals. The dashed lines in panels A,B mark the average capillary qO_2 and SR for the sham and CLP, respectively.
