# Supplementary material for: Sepsis impairs microvascular autoregulation and delays capillary response within hypoxic capillaries
Source: Crit Care. 2015 Nov 5;19:389. doi: 10.1186/s13054-015-1102-7 (PMC4634189; doi:10.1186/s13054-015-1102-7)
Supplement: Additional file 7: — Broader implications of impaired microvascular autoregulation in skeletal muscle. (PDF 50 kb) [file 13054_2015_1102_MOESM7_ESM.pdf]

Broader implications of impaired microvascular autoregulation in skeletal muscle

While the rat hind limb *extensor digitorum longus* (EDL) skeletal muscle was used in this study for technical reasons, our findings may apply to the heart and diaphragm as these muscular tissues share similar microvascular morphology with skeletal muscle, are known to develop similar microvascular derangements during sepsis [1, 2] and iNOS/NO upregulated in skeletal muscle [3] is reported to be negatively inotropic in the heart and diaphragm [4, 5]. This suggests that NO overproduction modulates both decreased contractility and cardiac depression in the septic heart and muscle weakness and respiratory failure in the septic lung. We hypothesize that a further common link between these tissues is sepsis induced impairment of RBC O_2-dependent ATP signaling with commensurate impaired microvascular autoregulation leading to mismatching of capillary oxygen delivery and local tissue oxygen consumption. Thus impaired erythrocyte O_2-dependent ATP signaling and associated microvascular dysfunction is hypothesized to be a general phenomenon. If this microvascular impairment exists to the same extent that it does in skeletal muscle, then it may help explain the upregulation of hypoxia-inducible factor (HIF-1α) and VEGF gene expression observed in the septic heart [1], as it responds to tissue hypoxia.

1. Bateman RM, Tokunaga C, Kareco T, Dorscheid DR, Walley KR: **Myocardial hypoxia-inducible HIF-1alpha, VEGF, and GLUT1 gene**

expression is associated with microvascular and ICAM-1 heterogeneity during endotoxemia. *American journal of physiology Heart and circulatory physiology* 2007, **293**(1):H448-456.

2. Boczkowski J, Vicaut E, Aubier M: **In vivo effects of Escherichia coli endotoxemia on diaphragmatic microcirculation in rats**. *Journal of applied physiology* 1992, **72**(6):2219-2224.
3. Bateman RM, Sharpe MD, Goldman D, Lidington D, Ellis CG: **Inhibiting nitric oxide overproduction during hypotensive sepsis increases local oxygen consumption in rat skeletal muscle**. *Critical care medicine* 2008, **36**(1):225-231.
4. Boczkowski J, Lanone S, Ungureanu-Longrois D, Danialou G, Fournier T, Aubier M: **Induction of diaphragmatic nitric oxide synthase after endotoxin administration in rats: role on diaphragmatic contractile dysfunction**. *The Journal of clinical investigation* 1996, **98**(7):1550-1559.
5. Herbertson MJ, Werner HA, Walley KR: **Nitric oxide synthase inhibition partially prevents decreased LV contractility during endotoxemia**. *The American journal of physiology* 1996, **270**(6 Pt 2):H1979-1984.
